# Light-Induced Thiol Oxidation of Recoverin Affects Rhodopsin Desensitization

**DOI:** 10.3389/fnmol.2018.00474

**Published:** 2019-01-07

**Authors:** Evgeni Yu. Zernii, Aliya A. Nazipova, Ekaterina L. Nemashkalova, Alexey S. Kazakov, Olga S. Gancharova, Marina V. Serebryakova, Natalya K. Tikhomirova, Viktoriia E. Baksheeva, Vasiliy I. Vladimirov, Dmitry V. Zinchenko, Pavel P. Philippov, Ivan I. Senin, Sergei E. Permyakov

**Affiliations:** ^1^Belozersky Institute of Physico-Chemical Biology, Lomonosov Moscow State University, Moscow, Russia; ^2^Institute of Molecular Medicine, Sechenov First Moscow State Medical University, Moscow, Russia; ^3^Institute for Biological Instrumentation of the Russian Academy of Sciences, Pushchino, Russia; ^4^Institute for Regenerative Medicine, Sechenov First Moscow State Medical University, Moscow, Russia; ^5^Shemyakin and Ovchinnikov Institute of Bioorganic Chemistry of the Russian Academy of Sciences, Pushchino, Russia

**Keywords:** light-induced retinal damage, photoreceptor, apoptosis, neuronal calcium sensor, recoverin, thiol oxidation, disulfide dimerization, GRK1

## Abstract

The excessive light illumination of mammalian retina is known to induce oxidative stress and photoreceptor cell death linked to progression of age-related macular degeneration. The photochemical damage of photoreceptors is suggested to occur via two apoptotic pathways that involve either excessive rhodopsin activation or constitutive phototransduction, depending on the light intensity. Both pathways are dramatically activated in the absence of rhodopsin desensitization by GRK1. Previously, we have shown that moderate illumination (halogen lamp, 1,500 lx, 1–5 h) of mammalian eyes provokes disulfide dimerization of recoverin, a calcium-dependent regulator of GRK1. Here, we demonstrate under *in vivo* conditions that both moderate long-term (metal halide lamp, 2,500 lx, 14 h, rat model) and intense short-term (halogen lamp, 30,000 lx for 3 h, rabbit model) illumination of the mammalian retina are accompanied by accumulation of disulfide dimer of recoverin. Furthermore, in the second case we reveal alternatively oxidized derivatives of the protein, apparently including its monomer with sulfinic group. Histological data indicate that thiol oxidation of recoverin precedes apoptosis of photoreceptors. Both disulfide dimer and oxidized monomer (or oxidation mimicking C39D mutant) of recoverin exhibit lowered α-helical content and thermal stability of their apo-forms, as well as increased Ca^2+^ affinity. Meanwhile, the oxidized monomer and C39D mutant of recoverin demonstrate impaired ability to bind photoreceptor membranes and regulate GRK1, whereas disulfide dimer exhibits notably improved membrane binding and GRK1 inhibition in absence of Ca^2+^. The latter effect is expected to slow down rhodopsin desensitization in the light, thereby favoring support of the light-induced oxidative stress, ultimately leading to photoreceptor apoptosis. Overall, the intensity and duration of illumination of the retina affect thiol oxidation of recoverin likely contributing to propagation of the oxidative stress and photoreceptor damage.

## Introduction

Photochemical damage is the most common form of the light-induced retinal damage. The prolonged eye exposure to sunlight or artificial light sources may cause PD of the retina, associated with progression of light-induced retinopathies (light maculopathy) (Organisciak and Vaughan, 2010; van Norren and Vos, 2016; Zernii et al., 2016). Being accumulated over years, photochemical injuries of the retina were suggested to provoke AMD, the major cause of blindness in the elderly worldwide (Loeffler et al., 2001; Plestina-Borjan and Klinger-Lasić, 2007; Vojniković et al., 2007). Furthermore, recent advances in ophthalmology and ophthalmic surgery increased incidence of iatrogenic PD of the retina caused by illumination of slit lamps, indirect ophthalmoscopes, fiberoptic endoilluminators and light sources of operative microscopes (Hunter et al., 2012; Wolffe, 2016). Thus, the establishment of molecular mechanisms underlying PD is an urgent task, as it will allow identifying approaches to the prevention and treatment of both iatrogenic and age-related retinal diseases.

Photoreceptor cells were recognized as primary targets of retinal PD. The damage is initiated in distal tips of their outer segments eventually engaging the entire cell (Organisciak and Vaughan, 2010; Hunter et al., 2012). There are several hypotheses of light-induced damage to photoreceptors, but they all consider rhodopsin as a key trigger for cell death pathways (Grimm et al., 2000; Organisciak and Vaughan, 2010). The main pathogenic factor is thought to be the reactive oxygen species, generated by the bleaching of rhodopsin. The light-induced oxidative stress results in oxidation of various molecules, including lipids and proteins, and ultimately leads to apoptosis of photoreceptors (Organisciak and Vaughan, 2010). Two major apoptotic pathways were suggested to become activated, depending on intensity of the light illumination. Low-intensity light induces constitutive transducin activation and excessive phototransduction that may activate PERK pathway of the unfolded protein response (Hao et al., 2002; Wang and Chen, 2014). By contrast, bright light induces apoptosis independently of transducin, but involves activation of transcription factor AP-1 (Hao et al., 2002). The specific mechanisms that trigger photoreceptor apoptosis pathways remain obscure, although they were suggested to depend on rhodopsin desensitization by GRK1 and arrestin (Chen et al., 1999a,b) and may include thiol oxidation of photoreceptor proteins (Lieven et al., 2012; Zernii et al., 2015b). Indeed, specific sulfur reductant protected photoreceptors from light-induced degeneration *in vivo* (Lieven et al., 2012).

The redox proteomic screening of retinal extracts identified appearance of disulfide homodimers of visual arrestin in response to toxic levels of light (Lieven et al., 2012). Visual arrestin terminates the phototransduction cascade by binding to phosphorylated light-activated rhodopsin (Gurevich et al., 2011). Hanson et al. revealed that oligomeric form of arrestin looses ability to bind the light-activated phosphorhodopsin (Hanson et al., 2007). Consistently, Lieven et al. (2012) demonstrated that disulfide dimers of visual arrestin generated in the retina during photic injury did not form stable complexes with its physiological binding partners, rhodopsin and enolase 1. Thus, disulfide dimerization of arrestin could contribute to light/oxidative stress-induced cell death pathways via affecting rhodopsin desensitization or activity of other arrestin targets (Song et al., 2006).

Recently, we reported that recoverin is one more photoreceptor protein undergoing light-induced disulfide dimerization under *ex vivo* and *in vivo* conditions (Zernii et al., 2015b). Recoverin is a Ca^2+^-sensor membrane-binding protein that serves as a Ca^2+^-dependent inhibitor of GRK1 in retinal rod cells. It coordinates two calcium ions by the second and the third EF-hand motifs thereby exposing its GRK1-recognizing site and N-terminal myristoyl group according to the mechanism known as a ‘Ca^2+^-myristoyl switch’ (for reviews, see Ames and Lim, 2012; Philippov and Zernii, 2012). Recoverin belongs to NCS proteins, which are expressed in the brain and retina where they transduce calcium signals in a wide range of signaling pathways and they are genetically linked to degenerative diseases (for reviews, see Burgoyne, 2007; Ames and Lim, 2012; Koch and Dell’Orco, 2015). A characteristic feature of the NCS family members is a highly conserved cysteine residue located in the third position of their first non-functional EF-hand motif (C39 in recoverin), which is sensitive to redox conditions. Recoverin was the first NCS protein that was shown to exhibit redox sensitivity of the thiol (Permyakov et al., 2007). Some of recoverin orthologs contain only one conservative cysteine (in mice, rats and cows), whereas the others include one more cysteine (in rabbits and humans). The conservative cysteine Cys39 of bovine recoverin undergoes oxidation under mild oxidizing *in vitro* conditions, forming a disulfide dimer and a thiol oxidized monomeric form (Permyakov et al., 2007). Recoverin mutant C39D that mimics oxidative conversion of Cys39 into sulfenic, sulfinic or sulfonic acids, exhibits decreased α-helicity and thermal stability, as well as suppressed affinity to photoreceptor membranes and GRK1 (Permyakov et al., 2012; Ranaghan et al., 2013). Disulfide dimer of recoverin as well as its multimeric/aggregated forms are accumulated in retina of the experimental animals subjected to moderate illumination of eyes with visible light (1,500 lx, 1–5 h), while monomeric recoverin remains mostly reduced (Zernii et al., 2015b). Histologic study demonstrates that the light-induced oxidation of recoverin occurs in intact retina and precedes damage of the photoreceptor layer. Taken together, these data suggest involvement of thiol oxidation of recoverin in triggering of photoreceptor cell damage/death mechanisms.

Here, we investigated the oxidative state of recoverin in mammalian retina exposed to various doses of visual light illumination such as long-term moderate illumination (2,500 lx for 14 h, rats without anesthesia) or short-term moderate/intense illumination (2,200/30,000 lx for 3 h, rabbits under general anesthesia). We found that both types of illumination promoted disulfide dimerization of recoverin. Meanwhile, in the rabbit model the oxidized derivatives of the protein included monomer with intramolecular disulfide bond (due to presence of the second Cys in rabbit recoverin) and monomer with sulfinic group. In both models, the oxidation of recoverin preceded apoptosis of photoreceptors. Of interest, disulfide dimerization and oxidation of monomeric recoverin caused similar changes in the protein secondary structure, its overall stability and Ca^2+^ affinity, but differently affected its functional properties. The oxidized monomer (or oxidation mimicking C39D mutant) of recoverin demonstrated impaired ability to bind photoreceptor membranes and regulate GRK1, whereas disulfide dimer of recoverin exhibited improved membrane binding and GRK1 inhibition in absence of Ca^2+^. We proposed that the latter effect would slow down rhodopsin desensitization in the light, which may favor retention of the light-induced oxidative stress and induction of photoreceptor apoptosis.

## Materials and Methods

### Illumination of Mammalian Eyes Under *in vivo* Conditions

Rat eye illumination was performed exactly as described in Novikova et al. (2014). Forty 2-month-old Wistar rats were used. Prior to the experiment, the animals were housed under normal vivarium conditions (12 h light/12 h dark cycle, 22–25°C, 55–60% humidity). Before illumination, rats were kept in the dark for 14 h for dark adaptation of their eyes. In experimental group, 16 unrestrained animals were exposed to visual light for 14 h using an NC-DE 70W/DW RX7s metal halide lamp (NARVA, Germany) with the following specifications: power 70 W, luminous flux 5,000–5,500 lumens, color temperature 4,000 K. The lamp was placed 2 m from the animal cages, yielding intensity of retinal illumination of 2,500 lx (0.003 W/cm^2^) and total dose of 151 J/cm^2^. In control group, eight animals were kept dark-adapted for the same time interval. The rats were decapitated under intraperitoneal anesthesia immediately after the illumination or at the lapse of 7 days during which the animals were kept under normal conditions described above.

Illumination of the rabbit eyes was performed according to the previously described procedure (Zernii et al., 2015b) with modifications. Thirty six healthy pigmented rabbits (6 months old, weight of 2.3–3 kg) were kept for 2 weeks at a 12 h light-dark cycle at temperature of 22–25°C and humidity of 55–60% with free access to food and water. Prior to experiment, all animals were dark-adapted for 12 h and then anesthetized by intramuscular injection of a commercial preparation containing 50 mg/ml tiletamine and 50 mg/ml zolazepam (15 mg of preparation per kg of body weight). One eye of each animal was exposed to visual light for 3 h using fiber-optic illuminator equipped with 150 W halogen lamp (Euromex Microscopen). The device settings were adjusted to ensure retina illuminance of 2,200 lx (0.011 W/cm^2^; illumination scheme 1) or 30,000 lx (0.15 W/cm^2^; illumination scheme 2) yielding total dose of 118 J/cm^2^ or 1620 J/cm^2^, respectively. Another eye was kept in the dark to be used as a control. The pupils of the light-exposed eyes were dilated using 25 mg/ml solution of phenylephrine hydrochloride. The animals were euthanized with an overdose of the anesthetic either immediately after the experiment, or after 3 days of normal husbandry.

Animal handling was performed according to the guidelines of the Association for Research in Vision and Ophthalmology for use of animals in ophthalmic and vision research (ARVO). The protocol was approved by the Belozersky Institute of Physico-chemical Biology Animal Care and Use Committee (Protocol number 1/2016).

### Identification of Recoverin Forms in Retinal Extracts

Detection and identification of monomeric and multimeric oxidized forms of recoverin in retinal extracts were performed following the protocols developed in Zernii et al. (2015b) with modifications. The retinas of the enucleated rat and rabbit eyes were isolated in the dim red light at 4°C and homogenized in 50 mM Tris-HCl buffer (pH 7.5), 100 mM NaCl on ice. The protein extracts were centrifuged at 39,000 × *g* (20 min, 4°C) and the supernatants were incubated for 1 h with rabbit polyclonal anti-recoverin antibodies, immobilized on CNBr-activated Sepharose 4B (GE Healthcare) in 0.1 M potassium phosphate buffer (pH 8.2), 150 mM NaCl at 4°C, with gentle shaking. The matrix was washed with 300 mM NaCl in the same buffer and recoverin forms were eluted with 200 mM sodium citrate buffer (pH 2.5), 150 mM NaCl, 5% glycerol. The resulting protein samples were adjusted to pH 6.2, concentrated and analyzed by non-reducing or reducing Western blotting using affinity-purified rabbit polyclonal anti-recoverin antibodies (Senin et al., 2011) or mouse monoclonal anti-recoverin antibodies (Santa Cruz Biotechnology), for rat and rabbit retinal extracts, respectively. The antigen-antibody complexes were visualized in the ChemiDoc^TM^ MP System (Bio-Rad) using Enhanced chemiluminescence (ECL) kit (Bio-Rad).

Alternatively, 5 μl aliquots of the immunoaffinity purified recoverin were desalted using P2 ZipTip C18 pipette tips, the protein was eluted by 50% acetonitrile with 0.1% TFA, and 0.5 μl of the resulting solution were mixed on a steel target with 0.5 μl of 20 mg/ml 2,5-dihydroxybenzoic acid in 30% (v/v) acetonitrile with 0.5% (v/v) TFA, and analyzed by mass spectrometry. For peptide mass fingerprinting, recoverin was digested by incubation with 5 μl of 15 μg/ml sequencing grade modified trypsin (Promega) in 0.05 M NH_4_HCO_3_ at 37°C for 1 h. The reaction mixture was adjusted by 10 μl of 0.5% TFA and 1 μl of the resulting solution was mixed on a steel target with 0.5 μl of 20 mg/ml 2,5-dihydroxybenzoic acid in 20% (v/v) acetonitrile with 0.5% (v/v) TFA, and subjected to mass spectrometry analysis. Mass spectra were recorded using an ultrafleXtreme MALDI-TOF/TOF mass spectrometer (Bruker Daltonics) equipped with a Smartbeam-II laser (Nd:YAG, 355 nm). The [MH]^+^ molecular ions of full-length proteins were analyzed in linear mode; the values of average m/z ratios were accurate to 10 Da. Peptide mass fingerprints were obtained in reflector mode. Monoisotopic [MH]^+^ molecular ions were measured in the 600–5,000 m/z range with a peptide tolerance of 30 ppm. MS/MS spectra of selected peptides were obtained using Lift mode with accuracy of 1 Da for daughter ion measurements. Mass spectra were analyzed using flexAnalysis 3.3 software (Bruker Daltonics). Protein analysis was carried out by MS and MS/MS ions searches using Mascot software (Matrix Science) and the NCBI protein database.

### Histologic Examination

For histological analysis, the enucleated rat and rabbit eyes were fixed in a Bouin’s (rats) or Carnoy’s (rabbits) solutions. After the fixation, the posterior segments containing retina, choroid, and sclera were isolated. The preparations were washed in ethanol and subjected to routine histological processing including dehydration by sevenfold incubation in absolute isopropanol for 5 h, and embedding of properly oriented samples into Histomix paraffin medium. Sagittal sections (4–7-μm thick each) were obtained from each paraffin block. The sections were mounted on slides, deparaffinized, rehydrated, stained with Carazzi’s hematoxylin and 0.5% eosin Y and examined using LEICA DM 4000B (Leica) and AxioScope A.1 (Carl Zeiss) microscopes. Microphotographs were obtained using AxioCam MRc5 high-resolution digital camera (Carl Zeiss) and Leica DFC400 digital camera (Leica). Processing of the microphotographs was performed using the AxioVision 8.0 (Carl Zeiss) and Adobe Photoshop CS6 Extended (Adobe Systems, United States) software.

### Preparation of Recoverin Forms

Recombinant myristoylated bovine recoverin (reduced monomer, RmRec) was prepared according to previously published procedure (Zernii et al., 2011). Recoverin mutant C39D was obtained as described in previous study (Permyakov et al., 2012). The degree of myristoylation of RmRec and C39D was determined by analytical HPLC using C18 “Symmetry” 3.9 × 150 mm reversed-phase column (Waters) and was more than 95%. Disulfide dimer (dRec) and oxidized monomer (OmRec) of recoverin were prepared from RmRec (Zernii et al., 2015b). Briefly, RmRec was incubated with 0.005% H_2_O_2_ at 30°C for 5 h and the resulting sample was subjected to gel-filtration using Superdex^TM^ 75 HR 10/30 HPLC column (GE Healthcare Life Sciences). The fractions containing dRec or OmRec were collected and dialyzed against ultrapure water at 4°C, freeze-dried and stored at -70°C.

For identification of oxidized derivatives of recoverin forming under mild oxidizing *in vitro* conditions, the recoverin sample was prepared mainly as described in previous study (Permyakov et al., 2007). Briefly, recombinant bovine recoverin was dialyzed overnight under non-reducing conditions against 50 mM Tris-HCl buffer (pH 7.5), 100 mM NaCl, followed by MS analysis.

Concentration of the recoverin forms was measured spectrophotometrically using a molar extinction coefficient at 280 nm of 24,075 M^-1^ cm^-1^, calculated according to Pace et al. (1995). For comparative studies, the concentration of dRec was calculated per mole of recoverin monomer.

### Calcium Binding Assay

Ca^2+^ binding to RmRec/dRec (40 μM/20 μM) was examined using ^45^Ca^2+^-binding assay (Weiergraber et al., 2006; Zernii et al., 2013). Briefly, recoverin was incubated with increasing ^45^Ca^2+^ concentrations, followed by the protein separation using ultrafiltration. Ca^2+^ concentrations were determined by radioactivity counting. Stoichiometry of Ca^2+^ binding was determined from the excess Ca^2+^ in the protein sample over that present in the ultrafiltrate. The resulting values were plotted versus [Ca^2+^]_free_ and the plots were fitted to the 4-parameter Hill equation using SigmaPlot 11 software (Systat Software). The apparent equilibrium dissociation constant (*K*_D_) and Hill coefficient values characterizing Ca^2+^ affinity of recoverin were obtained from these fits.

### Fluorescence Measurements

Fluorescence emission spectra were measured with a Cary Eclipse spectrofluorimeter (Varian Inc.), equipped with a Peltier-controlled cell holder essentially as described before (Baksheeva et al., 2015). Protein concentration was 6–14 μM. Measurements were carried out in 10 mM HEPES-KOH, 150 mM KCl, pH 7.3 buffer in the presence of either 1 mM CaCl_2_ or 1 mM EGTA and 1 mM DTT for RmRec and C39D recoverin. Concentrations of bis-ANS and protein in these experiments were 1 μM and 6 μM, respectively. The concentration of water stock solution of bis-ANS was evaluated using molar extinction coefficient 𝜀_385nm_ of 16,790 M^-1^cm^-1^ (Farris et al., 1978). Fluorescence of bis-ANS/recoverin was excited at 385/280 nm; emission slit width was 5 nm. All spectra were fitted to log-normal curves (Burstein and Emelyanenko, 1996) using LogNormal software (IBI RAS, Pushchino, Russia), implementing non-linear regression algorithm by Marquardt (Marquardt, 1963). The fluorescence spectrum maximum positions (λ) were obtained from these fits. Spectrofluorimetric temperature scans were performed stepwise, allowing the sample to equilibrate at each temperature. Temperature was monitored inside the sample cell. The average heating rate was 0.5°C/min. The *T*_1/2_ were calculated from temperature dependence of λ using Boltzmann sigmoid as implemented in OriginPro 8.0 (OriginLab Corporation) software for Ca^2+^-loaded recoverin, and as described in Permyakov and Burstein (1984) for apo-forms.

### Circular Dichroism Measurements

Circular dichroism measurements were carried out with a JASCO J-810 spectropolarimeter (JASCO Inc., Japan), equipped with a Peltier-controlled cell holder as previously described (Zernii et al., 2015a). Protein concentration was 4 μM for RmRec and OmRec, or 2 μM for dRec. The contribution of buffer [pH 8.2, 10 mM H_3_BO_3_–KOH, 1 mM CaCl_2_ or 1 mM EDTA, 20 μM DTT (for RmRec)] was subtracted from the experimental spectra. Estimations of the secondary structure fractions were made using the CDPro software package (Sreerama et al., 2000).

### The Equilibrium Centrifugation Assay of Membrane Binding

Bovine ROS and urea-washed photoreceptor membranes were prepared from frozen retinas according to a method described in Grigoriev et al. (2012). The binding of recoverin forms to photoreceptor membranes was performed using equilibrium centrifugation assay (Permyakov et al., 2003), with modification described in Zernii et al. (2003). Samples of RmRec (30 μM) or dRec (15 μM) were mixed with bleached urea-washed photoreceptor membranes containing 0–100 μM rhodopsin in 20 mM Tris–HCl (pH 8.0), 150 mM NaCl, 20 mM MgCl_2_, 1 mM DTT (for RmRec), with addition of Ca^2+^/5,5′Br2-BAPTA buffer yielding 0.11–500 μM free [Ca^2+^] in total volume of 50 μl. The probes were incubated at 37°C for 20 min and centrifuged (15 min, 14,000 rpm). The supernatants were discarded and the pellets were dissolved in 50 μl of sample buffer [125 mM Tris-HCl, pH 6.8, 4% (w/v) SDS, 20 (v/v) glycerol, 10% (v/v) b-mercaptoethanol, 0.004% (w/v) bromophenol blue] and analyzed by SDS-PAGE. The amounts of recoverin forms bound to photoreceptor membranes were evaluated by densitometric scanning of the corresponding bands in polyacrylamide gel.

### Rhodopsin Phosphorylation Assay

GRK1 (rhodopsin kinase) was purified from ROS as described elsewhere (Senin et al., 2011). GRK1 assay was performed as described in Vladimirov et al. (2018). Briefly, 40 μM RmRec or C39D mutant, or 20 μM dRec were mixed with 10 μM rhodopsin (urea-washed photoreceptor membranes) and 0.3–0.5 units of GRK1 in 20 mM Tris-HCl (pH 8.0), 100 mM NaCl, 1 mM [γ-^32^P]ATP, 1 mM DTT, 3 mM MgCl_2_, with addition of Ca^2+^/5,5′Br_2_-BAPTA buffer yielding either 200 μM or 0.01 μM free [Ca^2+^]. Free Ca^2+^-concentration in the buffers was calculated using Webmaxc Standard software (Stanford University). The reaction (15 min) was initiated by light illumination and terminated by addition of sample buffer for SDS-PAGE. The proteins were separated by polyacrylamide gel electrophoresis and ^32^P emission was registered by phosphorimaging radioautography.

### Surface Plasmon Resonance Studies

N-terminal domain of rhodopsin kinase (N-GRK1) and its fragment M1-S25 (1-25GRK1) fused with glutathione-S-transferase were obtained according to previously published procedure (Grigoriev et al., 2012). SPR measurements were performed at 25°C using Bio-Rad ProteOn^TM^ XPR36 protein interaction array system (Kazakov et al., 2017). Ligand (40 μg/mL N-GRK1 or 1-25GRK1 in 10 mM sodium acetate, pH 4.5 buffer) was immobilized on ProteOn GLH sensor chip surface (up to 10,000–15,000 resonance units, RUs) by amine coupling, according to the manufacturer’s instructions. The remaining activated amine groups on the chip surface were blocked by 1 M ethanolamine solution. Analyte (RmRec, OmRec, C39D and dRec, 1 μM to 40 μM) in a running buffer (10 mM HEPES, 150 mM NaCl, 0.05% TWEEN 20, pH 7.4 buffer with 1 mM CaCl_2_ and 2 mM MgCl_2_) was passed over the chip at a rate of 30 μl/min for 350 s, followed by flushing the chip with the running buffer for 1,500 s. The double-referenced SPR sensograms were globally fitted according to a heterogeneous ligand model, which assumes existence of two populations of the ligand (L1 and L2) that bind a single analyte molecule (A):

(1) $$\begin{array}{c} \text{Kd1}\\ L1\;+\;A↔\\ \text{Kd1} \end{array}L1A;L2\;+\;\begin{array}{c} \text{Kd2}\\ A↔L2A\\ \text{Kd2} \end{array}$$

where *Kd* and *kd* refer to equilibrium and kinetic dissociation constants, respectively. *Kd, kd* and *Rmax* (maximum response) values were evaluated using Bio-Rad ProteOn Manager^TM^ v.3.1 software. The sensor chip surface was regenerated by passage of 0.5% SDS water solution for 50 s.

## Results

### Illumination of Mammalian Retina Promotes Accumulation of Disulfide Dimer of Recoverin

To explore redox state of recoverin (both for the protein containing one cysteine and the one with two cysteines) in mammalian retina exposed to different doses of illumination, two animal models were used. In the first model, unrestrained rats were exposed to long-term (14 h) illumination by visual light of moderate intensity (metal halide lamp, 2,500 lx, 0.003 W/cm^2^). In the referent group, rats were kept dark-adapted for the same time intervals. The second model employed pigmented rabbits under general anesthesia that were exposed to short-term (3 h) illumination by visual light of either moderate (halogen lamp, 2,200 lx, 0.011 W/cm^2^; scheme 1) or high intensity (halogen lamp, 30,000 lx; 0.15 W/cm^2^; scheme 2). In this case, the illumination doses were chosen to model iatrogenic retinal damage induced by common operative microscopes used in surgical ophthalmology (see the section “Discussion”). One eye of each animal was illuminated, whereas another eye was kept in the dark for use as a reference. Immediately after the illumination/dark adaptation the animals were euthanized, the retinas were isolated, and soluble proteins were extracted from the isolated retinas. To improve identification of recoverin forms and their mass-spectrometry analysis (see below), the protein was quickly purified from the retinal extracts using immunoaffinity chromatography (Zernii et al., 2015b).

**FIGURE 1:**
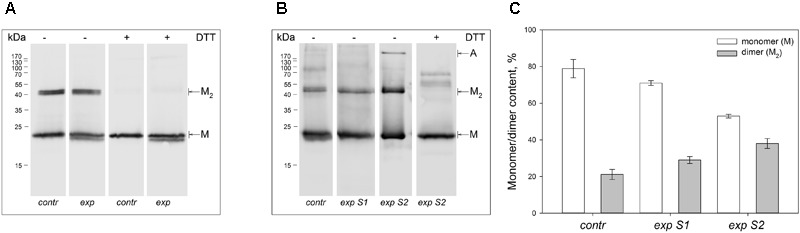
Identification of multimeric oxidized forms of recoverin in the retina of albino rats and pigmented rabbits exposed to different doses of visual light illumination. Western blotting under non-reducing or reducing conditions of recoverin fractions extracted from the rat retinas illuminated *in vivo* for 14 h with metal halide lamp (2,500 lx, ‘exp’) **(A)**, and rabbit retinas illuminated *in vivo* for 3 h with halogen lamp following scheme 1 (2,200 lx, ‘exp S1’) or scheme 2 (30,000 lx, ‘exp S2’) **(B)**. The recoverin fractions obtained from the dark-adapted retinas were used as a control (‘contr’). ‘M,’ ‘M2,’ and ‘A’ denote monomeric, dimeric, and multimeric/aggregated forms of recoverin, respectively. Volumes of the loaded recoverin samples were chosen to ensure nearly equivalent bands of the monomeric protein. The numbers in left-hand columns indicate the molecular masses of protein markers in kDa. **(C)** The weight fractions of monomeric and dimeric forms of rabbit recoverin estimated from the Western blotting data from at least three independent *in vivo* experiments.

Non-reducing Western blotting revealed recoverin dimer in the protein fractions extracted from all illuminated eyes of the both species, as indicated by appearance of the band of *ca* 45 kDa in addition to the band of recoverin monomer (*ca* 23 kDa, Figure 1). The dimeric recoverin was also found in control samples, but the dimer to monomer ratio was lower. In rabbit model illumination dose-dependent accumulation of the dimeric recoverin was observed (Figure 1B). Thus, weight fraction of the dimer was ∼20% in control animals and it increased up to ∼30% upon illumination following scheme 1 and up to ∼40% upon illumination following scheme 2 (Figure 1C). In the latter case, an additional band corresponding to multimeric/aggregated forms of the protein (>170 kDa) was identified, in accord with our previous findings (Zernii et al., 2015b) (Figure 1B). Meanwhile, this band was absent in the samples derived from the eyes illuminated according to the scheme 1, as well as in the control samples. Furthermore, such band was absent in the rat recoverin samples (Figure 1A). Since Western blotting of the samples from the both species under reducing conditions revealed disappearance of the dimer and multimeric bands, they were stabilized by disulfide bonds. It should be noted, that some bands of monomeric/dimeric recoverin exhibited bifurcation, which can be attributed to partial proteolysis of the protein or presence of its non-myristoylated form.

### Illumination of Rabbit Retina Induces Formation of Monomeric Thiol Oxidized Forms of Recoverin

The oxidative modifications of monomeric recoverin, extracted from the illuminated retinas, were explored by MALDI-TOF mass-spectrometry. The analysis was first performed using linear mode of the spectrometer for detection of [MH]^+^ molecular ions of full-size protein, enabling mass determination with accuracy of 10 Da. In MS spectra of recoverin samples from the illuminated rat eyes only one peak with m/z ratio of 23,483 Da was observed within the 23,000–24,000 m/z range (data not shown). Despite a slight difference from expected mass of the intact recoverin (23,472 Da), this peak corresponded to rat recoverin, as confirmed by peptide mass fingerprinting (Supplementary Table S1). Two C39-containing peptides (molecular masses of 2507.1666 Da and 3817.8752 Da) were identified in trypsin hydrolysate of the protein. Since C39 residue of the both peptides remains reduced, the illumination of rat eyes by visual light of moderate intensity is accompanied by accumulation of disulfide dimer of recoverin, whereas the conservative thiol of the monomeric protein remains intact.

**FIGURE 2:**
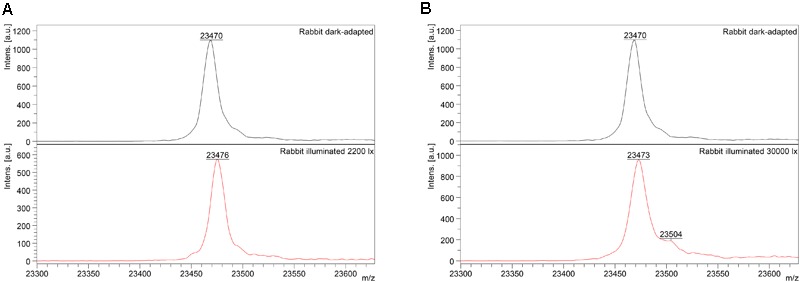
Identification of monomeric oxidized forms of recoverin in the retina of pigmented rabbits exposed to different doses of visual light illumination. MALDI-TOF/TOF mass spectra of monomeric recoverin ([MH]^+^ molecular ions of full-length proteins) extracted from the rabbit retinas illuminated *in vivo* for 3 h with halogen lamp following scheme 1 (2,200 lx) **(A)** or scheme 2 (30,000 lx) **(B)**.

Mass-spectrometry of recoverin derived from the referent (dark-adapted) rabbit eyes revealed a single peak with m/z ratio of 23,470–23,475 Da that corresponded to the fully reduced protein (Figure 2). Meanwhile, MS analysis of recoverin samples obtained upon the high-intensity illumination of rabbit eyes (scheme 2) additionally revealed a minor peak centered at m/z ratio of 23,504 Da (Figure 2B). This peak can be attributed to rabbit recoverin with two extra oxygen atoms. One of the possibilities is Cys39 oxidation with formation of sulfinic acid. Although we failed to detect this derivative by direct MS/MS evidences, we confirmed its formation in recombinant bovine recoverin under mild oxidizing *in vitro* conditions using mass fingerprinting of its trypsin hydrolysate peptides. Two cysteine-containing peptides of this protein, E38-R43 and F23-R43, were found in reduced (648.3026 and 2523.1568 Da), singly oxidized (664.2945 and 2539.1372) and doubly oxidized (680.2717 and 2555.1170) states, thereby indicating conversion of Cys39 into sulfenic/sulfinic acid. The structure of these peptides was confirmed by MS/MS analysis (Supplementary Figure S1).

Interestingly, the peak at 23,504 Da was absent in the MS spectra of recoverin extracted from the animals exposed to lower dose of illumination within the scheme 1 (Figure 2A). Instead, mass fingerprinting of trypsin hydrolysate peptides revealed (MS and MS/MS analysis) recoverin form with an intramolecular disulfide bridge (opposite to rat recoverin, its rabbit ortholog contains two cysteine residues, C29 and C39). Among 20 identified peptides that cover 87% of the protein sequence (Supplementary Table S2) two peptides (molecular masses of 648.2951 Da and 1923.8839 Da) contained thiols of C29 and C39 residues in a reduced form. Meanwhile, the MS peak with m/z ratio of 2551.1380 corresponded to the peptide F23-R43, in which C29 and C39 residues lacked hydrogen atoms, i.e., formed an intramolecular disulfide bridge. Notably, neither of other redox-sensitive amino acids (M, W or H) were found in oxidized state, despite their presence in the identified peptides. Hence, C39 has the highest redox sensitivity among all recoverin residues, in line with our previous findings (Permyakov et al., 2007).

Overall, the accumulation of disulfide dimer of recoverin in the light-exposed rabbit eyes was accompanied by additional formation of oxidized monomer of the protein, which contained either an intramolecular disulfide bond or sulfinic acid, depending on illumination dose.

### Thiol Oxidation of Recoverin in Illuminated Mammalian Retina Precedes Apoptosis of Photoreceptors

**FIGURE 3:**
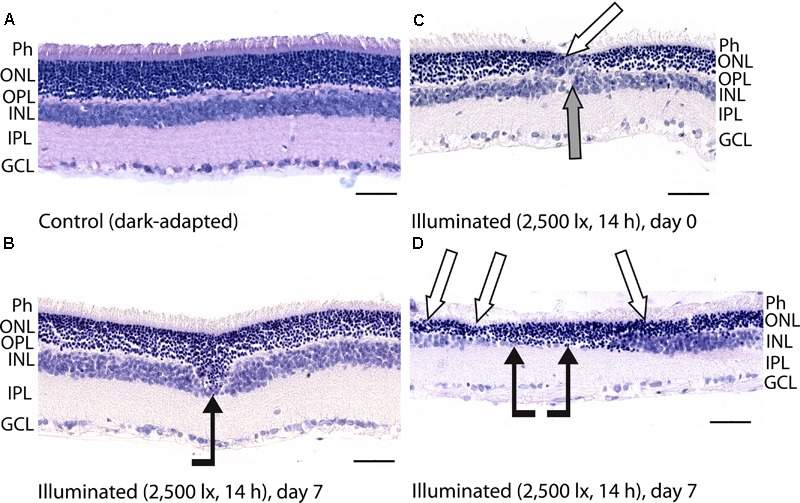
Histological analysis of posterior segments of the dark-adapted and illuminated *in vivo* rat eyes. Representative paraffin-embedded sections of the eye tissues were stained with hematoxylin and eosin (magnification 400×, scale bar 50 μm). Designations are as follows: GCL, ganglion cell layer; IPL, inner plexiform layer; INL, inner nuclear layer; OPL, outer plexiform layer; ONL, outer nuclear retinal layer; Ph, photoreceptor layer. **(A)** The retina of a control dark-adapted animal. **(B)** The retina isolated immediately after illumination for 14 h with metal halide lamp (2,500 lx). ONL migration in the vitreal direction is indicated by black arrow. **(C,D)** The retina isolated 7 days after the light exposure. ONL disorganization and thinning are indicated with white arrows. INL migration and thinning of OPL and INL are marked on **(C,D)** with gray and black arrows, respectively.

**FIGURE 4:**
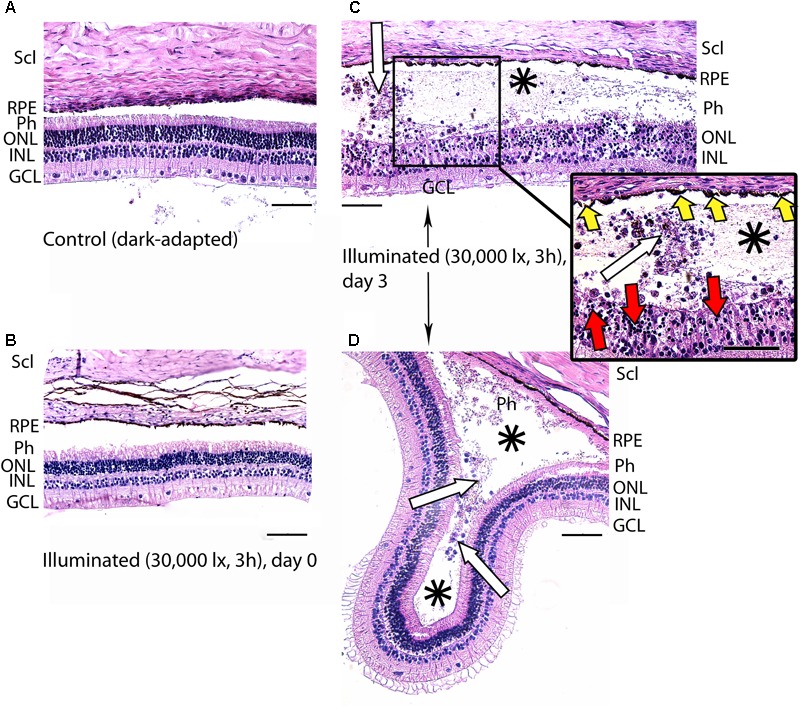
Histological analysis of posterior segments of the dark-adapted and illuminated *in vivo* rabbit eyes. Representative paraffin-embedded sections of the eye tissues were stained with hematoxylin and eosin (magnification 200×/400× on the inset; scale bar 20 μm). Designations are as follows: GCL, ganglion cell layer; INL, inner nuclear layer; ONL, outer nuclear layer; Ph, photoreceptor layer; RPE, retinal pigment epithelium; Scl, sclera. **(A)** Intact retina of a control dark-adapted animal. **(B)** The retina isolated immediately after illumination for 3 h with halogen lamp (30,000 lx). **(C,D)** The retina isolated 3 days after the light exposure. Macrophage-like cells in the space between RPE and the retina are indicated with white arrows. Retinal detachments from RPE are labeled with asterisks. Signs of karyopyknosis in ONL and changes in RPE are marked with red and yellow arrows, respectively **(C**, inset**)**.

To examine if the light-induced thiol oxidation of recoverin was accompanied by PD of mammalian retina, the illuminated/dark-adapted *in vivo* rat and rabbit eyes were analyzed by histological techniques. Posterior segments (containing RPE and neural retina) of the eyes were obtained upon decapitation of the animals either immediately after exposure to light or 3–7 days later. The samples were embedded in paraffin and stained using hematoxylin and eosin (Figures 3, 4).

Posterior segments of the rat eyes enucleated immediately after the prolonged light exposure exhibited multifocal changes in the retina affecting mainly photoreceptor layer without significant alterations in RPE (Figure 3). Although the overall structure of the retina remained intact, in some regions the signs of advanced stage of apoptosis such as karyopyknosis and formation of apoptotic bodies were observed. In addition, the retina exhibited thinning of the outer plexiform layer formed by photoreceptor axons, and shifting of the photoreceptor cell bodies in the vitreal direction (Figure 3B). Consistently, in 7 days after the illumination, the outer nuclear layer and inner nuclear layer of the rat retinas drastically decreased in thickness. Destruction of these layers together with deterioration of the outer plexiform layer resulted in severe changes in retina cytoarchitecture representing symptoms of irreversible retinal damage through apoptosis (Figures 3C,D).

In the rabbit samples prepared immediately after the light illumination following scheme 1 (data not shown) and scheme 2 (Figure 4B), the tissues of the eye fundus exhibited no changes as compared to referent eyes (Figure 4A). In the first case, the normal morphology of the retina was maintained even on the third and the seventh day of post-exposure (data not shown). Meanwhile, in the second case, the retina exhibited destructive changes on the third day of post-exposure and these changes involved both photoreceptor layer and RPE (Figures 4C,D). In the most affected regions of this tissue both outer and inner segments of photoreceptors were absent and multifocal death of photoreceptor cells was evident (Figure 4C, inset). Outer nuclear layer was sparse and photoreceptor cells showed morphological signs of apoptotic type cell death such as karyopyknosis (Figure 4C, inset, red arrows). These changes in outer nuclear layer were accompanied by cell death in inner nuclear layer of the retina resulting in destruction and thinning of these layers as well as outer plexiform layer lying between them. Furthermore, the damage involved RPE cells that were vacuolized, activated and increased in size (demonstrated «tombstone» morphology) (Figure 4C, inset, yellow arrows) and some of them migrated to the dying retinal cells (Figure 4C). Finally, the retina exhibited detachments from RPE, which were noticeable not only in the most photosensitive regions (Figure 4D), but also adjacent to the damaged areas (Figures 4C,D, asterisks), where the photoreceptors were not directly affected. In the newly formed wide space between RPE and outer nuclear layer, the residuals of photoreceptor segments became engulfed by macrophages-like cells (Figure 4C, inset, white arrows). These cells had pigment granules suggesting that they migrated from RPE. The presence of the macrophage-like cells in the space between RPE and the retina (Figure 4D, white arrows) reflected development of compensatory processes in the retina.

Thus, despite some differences in time scales, both rat and rabbit models of the light-induced retinal damage were characterized by death of photoreceptor cells via apoptosis, which developed in the post-exposure period. Since recoverin was isolated from the retinas immediately after the illumination, one can conclude that the revealed formation of disulfide dimer and oxidized monomeric forms of the protein precedes the apoptosis of photoreceptors.

### Disulfide Dimerization Increases Sensitivity of Recoverin to Calcium

To examine functional consequences of the light-induced thiol oxidation of recoverin, we studied *in vitro* Ca^2+^-dependent structural and functional properties of the following derivatives of the protein: reduced monomer (RmRec), disulfide dimer (dRec) and C39D mutant imitating conversion of C39 thiol into the negatively charged sulfinic acid. In some experiments, oxidized monomer of recoverin (OmRec) prepared by incubation of RmRec with 0.005% H_2_O_2_ was employed for comparison with C39D mutant that had been partially characterized in previous studies (Permyakov et al., 2012). The monomer of rabbit recoverin with intramolecular disulfide bond was left beyond the scope of our studies, as C29 residue of the protein is not conservative.

**FIGURE 5:**
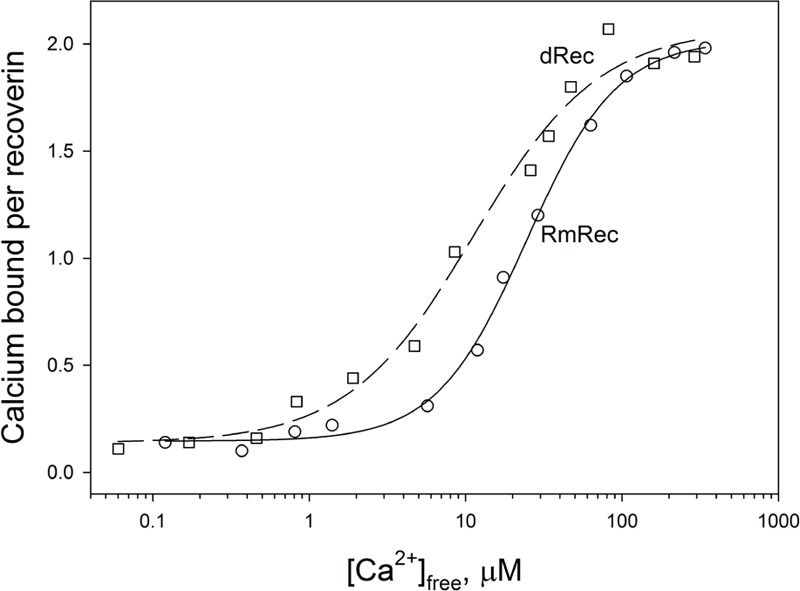
Calcium binding to RmRec/dRec. 40 μM RmRec (open circles) or 20 μM dRec (open squares) were incubated with increasing Ca^2+^ concentrations in the presence of ^45^Ca^2+^ and the Ca^2+^-bound protein was separated by ultrafiltration. Stoichiometry of Ca^2+^ binding determined from the excess Ca^2+^ in the protein sample over that present in the ultrafiltrate was plotted versus [Ca^2+^]_free_ and the plots were fitted to the 4-parameter Hill equation. Solid and dashed curves represent the best fits for RmRec and dRec, respectively.

Since recoverin is known to serve as both Ca^2+^ sensor and Ca^2+^ buffer in the cell (Makino et al., 2004), we explored the oxidation-induced changes in its Ca^2+^-binding parameters. According to ^45^Ca^2+^-binding assay, RmRec coordinated two Ca^2+^ ions in a cooperative manner with Hill coefficient of 1.5 and half-saturation at 24 μM (Figure 5 and Table 1), in accord with previous estimates (Zernii et al., 2011). Meanwhile, dRec exhibited markedly less cooperative Ca^2+^ binding (Hill coefficient of 1.1), although its stoichiometry remained unchanged. Furthermore, disulfide dimerization increased overall Ca^2+^ affinity of recoverin up to *K*_D_ value of 11 μM (Figure 5 and Table 1). Since the C39D mutant also demonstrated slightly increased affinity to Ca^2+^ (Permyakov et al., 2012), we concluded that both disulfide dimerization and conversion of Cys39 into sulfinic acid enhanced calcium sensitivity of recoverin, thereby affecting its Ca^2+^-dependent functions.

**Table 1:** The parameters of Ca^2+^-binding, thermal denaturation and membrane association of the recoverin variants.

Protein	Binding of Ca^2+^		Thermal stability		Binding to photoreceptor membranes	
	*K*_D_, μM^∗^ (*K*_A_^1^/*K*_A_^2^, 1/M^∗∗^)	Hill coefficient^∗^	*T*_1/2_, °C (1 mM EGTA)	*T*_1/2_, °C (1 mM CaCl_2_)	EC_50_, [Ca^2+^]_f_, μM^∗∗∗∗^	Hill coefficient^∗∗∗∗^
RmRec	24.5 (0.7 × 10^6^/0.23 × 10^6∗∗∗^)	1.50	65.8 (67.8^∗∗∗^)	76.5 (87.5^∗∗∗^)	3.4 (3.5^∗∗∗^)	1.8 (2.0^∗∗∗^)
C39D	-(1.5 × 10^6^/0.34 × 10^6∗∗∗^)	-	60.5^∗∗∗^	84.3^∗∗∗^	-(11.0^∗∗∗^)	-(1.6^∗∗∗^)
OmRec	-	-	59.3	77.2	–	-
dRec	11.2	1.1	46.6	73.0	5.1	5.3

*^∗^According to ^*45*^Ca^2+^-binding assay data fitted to the Hill model. ^∗∗^Estimated from spectrofluometric measurements within the model of sequential filling of Ca^2+^-binding sites as described in Permyakov et al. (2012). ^∗∗∗^The data from Permyakov et al. (2012). ^∗∗∗∗^According to equilibrium centrifugation assay data fitted to the Hill model.*

### Thiol Oxidation Reduces Structural Stability of Recoverin

**FIGURE 6:**
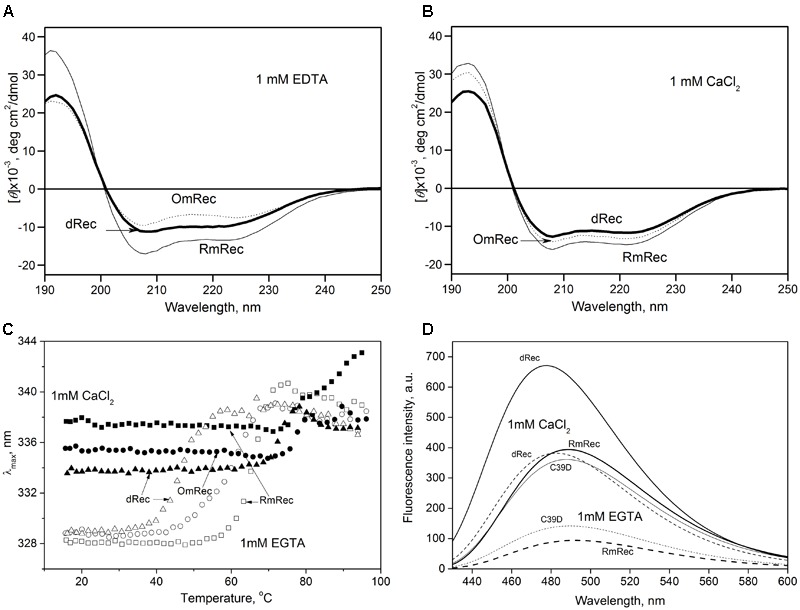
Structural properties of recoverin forms. **(A,B)** Far-UV CD spectra of Ca^2+^-free **(A)** or Ca^2+^-loaded **(B)** RmRec (4 μM, solid curves), OmRec (4 μM, dotted curves) and dRec (2 μM, thick solid curves) at 20°C, pH 8.2. **(C)** Thermal dependencies of fluorescence spectrum maximum position for Ca^2+^-free (open symbols) and Ca^2+^-loaded (filled symbols) RmRec (14 μM, squares), OmRec (14 μM, circles) and dRec (7 μM, triangles) samples at pH 7.3. **(D)** The binding of bis-ANS (1 μM) to Ca^2+^-free (dashed curves) or Ca^2+^-loaded (solid curves) RmRec (6 μM, medium curves), C39D mutant (6 μM, thin curves) and dRec (3 μM, thick curves) monitored by fluorescence emission spectrum of the dye at 20°C, pH 7.3.

To reveal an impact of thiol oxidation on recoverin structure, we examined physicochemical properties of RmRec, dRec, and OmRec and analyzed them with the respective data obtained earlier for C39D mutant (Permyakov et al., 2012). In absence of calcium dRec and OmRec exhibited decrease in negative molar ellipticity at 205–240 nm, as compared to RmRec (Figure 6A). Analysis of the CD spectra revealed decreased α-helicity of apo-dRec/OmRec, compensated by increase in the content of other elements of their secondary structure (Table 2). Calcium binding does not induce marked changes in secondary structure of RmRec, but shifts secondary structure fractions of OmRec and dRec toward those for RmRec (Figure 6B). Overall, α-helical content of recoverin decreased regardless of Ca^2+^ level in the order RmRec > C39D > OmRec > dRec. Thus, thiol oxidation disorganized secondary structure of recoverin, especially in the case of disulfide dimer.

**Table 2:** The secondary structure fractions for the recoverin forms estimated from the far-UV CD data shown in Figures 6A,B using CDPro software package (Sreerama et al., 2000).

Protein	α-Helices, %	β-Sheets, %	Turns, %	Unordered structure, %
*Apo-form*			
RmRec^∗^	54.6	9.2	13.6	22.5
C39D^∗^	49.2	11.0	15.0	24.7
OmRec	38.5	16.1	16.6	28.7
dRec	38.3	17.3	17.6	26.6
*Ca^2+^-loaded protein*			
RmRec^∗^	54.2	8.6	15.0	21.7
C39D^∗^	50.9	10.1	14.0	24.7
OmRec	50.2	12.7	13.5	23.7
dRec	43.9	12.6	16.7	26.8

*^∗^The data from Permyakov et al. (2012).*

Thermal unfolding of recoverin was monitored by changes in maximum position of tryptophan fluorescence spectrum (λ), which reflects solvent accessibility of its emitting Trp residues: protein unfolding induces red shift of its fluorescence spectrum maximum (Figure 6C). Since at temperatures below the thermal transition the λ values of Ca^2+^-bound forms of dRec and OmRec were lowered with respect to RmRec, the oxidized recoverin derivatives exhibited lower solvent exposure of emitting Trp residues. Calcium removal further protected them from the solvent, as observed for RmRec. In the absence of calcium the recoverin derivatives exhibited lowered thermal stability. Their *T*_1/2_ values decreased in the order observed for their α-helices fractions: RmRec > C39D > OmRec > dRec (Table 1). Calcium binding markedly enhanced thermal stability of all recoverin variants studied.

Finally, the oxidation-induced alterations in recoverin structure were examined by spectrofluorimetric monitoring of its interaction with bis-ANS, a hydrophobic probe recognizing surface-accessible non-polar protein cavities (Figure 6D) (Hawe et al., 2008). Fluorescence intensity of bis-ANS in the presence of RmRec was threefold to fivefold higher for the Ca^2+^-loaded protein, apparently reflecting Ca^2+^-induced exposure of the hydrophobic pocket of recoverin responsible for its interaction with GRK1 (Ames et al., 2006; Zernii et al., 2015a). In the presence of C39D mutant, bis-ANS exhibited fluorescence spectra similar to those observed for RmRec, which indicate equivalent surface hydrophobicities of these forms. Meanwhile, in the presence of disulfide dimer of recoverin bis-ANS showed notably increased fluorescence intensity as compared to RmRec. Thus, Ca^2+^-bound dRec exhibited highest surface hydrophobicity among the recoverin derivatives. Furthermore, surface hydrophobicity of Ca^2+^-free dRec resembled that of Ca^2+^-bound RmRec, which suggest that at low intracellular calcium levels dRec might expose the non-polar residues interacting with GRK1, and thereby could be aberrantly active.

### Disulfide Dimer of Recoverin Interacts With Photoreceptor Membranes and Inhibits Rhodopsin Kinase at Low Calcium

**FIGURE 7:**
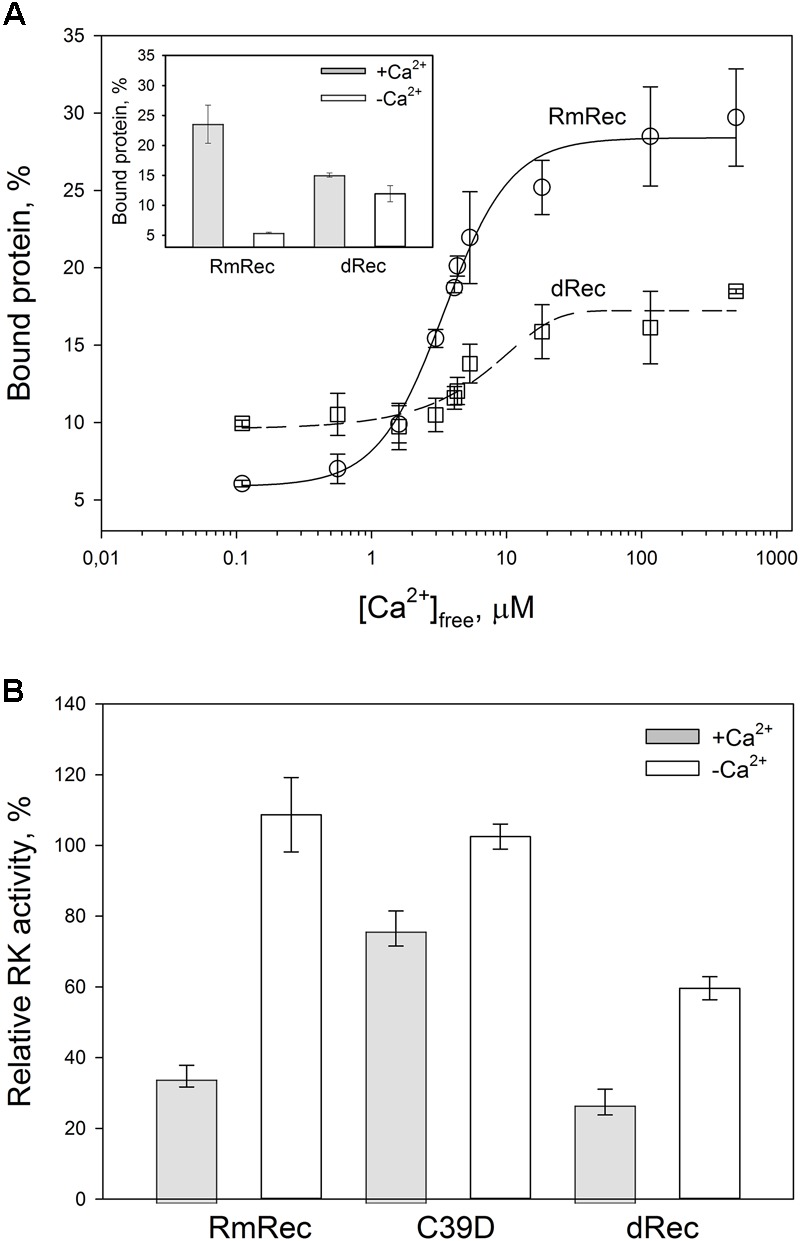
Functional properties of the recoverin forms. **(A)** The binding of 30 μM RmRec (open circles) and 15 μM dRec (open squares) to photoreceptor membranes. Recoverin was mixed with urea-washed photoreceptor membranes in the presence of 0.11–500 μM [Ca^2+^]_free_ at 37°C, pH 8.0 and the membranes were separated by ultracentrifugation. The fractions of membrane-bound protein evaluated by SDS-PAGE were plotted versus [Ca^2+^]_free_ and the plots were fitted to the 4-parameter Hill equation. Solid and dashed curves represent best fits for RmRec and dRec, respectively. The inset: fractions of the Ca^2+^-free and Ca^2+^-saturated protein bound to the membranes. **(B)** Inhibition of GRK1 by RmRec or C39D mutant (40 μM), or dRec (20 μM). Rhodopsin phosphorylation by GRK1 in the presence of [γ -^32^P]ATP was monitored at high [Ca^2+^]_free_ (200 μM Ca^2+^, filled bars) or low [Ca^2+^]_free_ (0.01 μM, open bars) by phosphorimaging radioautography.

Given the observed changes in Ca^2+^ affinity and surface hydrophobicity of recoverin induced by its disulfide dimerization, we suggested that membrane-binding properties of the thiol-oxidized protein could be affected. To probe this suggestion, we monitored association of dRec with photoreceptor membranes using equilibrium centrifugation assay. At saturating Ca^2+^ levels, the photoreceptor membrane-bound protein fraction for dRec was lowered with regard to RmRec by 50% (Figure 7A). This effect was accompanied by the respective shift in Ca^2+^ dependence of the membrane binding toward higher calcium concentrations (Figure 7A): the membrane binding of dRec is half-maximal at 5.1 μM free Ca^2+^ versus 3.4 μM free Ca^2+^ for RmRec (Table 1). In this respect, dRec resembled the C39D mutant that exhibited the half-maximal effect at 11 μM free Ca^2+^ (Table 1) (Permyakov et al., 2012). However, in the absence of Ca^2+^ dRec and C39D mutant demonstrated different behavior. Whereas the mutant exhibited low affinity to membranes similarly to RmRec (Permyakov et al., 2012), membrane binding of dRec increased about twofold as compared to RmRec (Figure 7A, inset). Taken together with the results of bis-ANS fluorescence studies (see Figure 6D), these data suggested that disulfide dimerization of recoverin abolishes Ca^2+^-myristoyl switch leading to constitutive exposure of its hydrophobic surfaces that anchor the protein on membrane at low calcium. It should be added that treatment of dRec with excess of DTT yielded monomeric recoverin, which bound to the membranes in Ca^2+^-dependent manner similarly to RmRec (∼26% and 5% of the control at high and low calcium, respectively).

Since Ca^2+^-myristoyl switch of recoverin involves exposure of its hydrophobic residues responsible for interaction with GRK1, we supposed that disulfide dimerization may affect regulatory activity of recoverin toward the enzyme. Therefore, we compared the effect of various recoverin forms on GRK1 activity in rhodopsin phosphorylation assay employing urea-washed photoreceptor membranes harboring the receptor. At high calcium concentrations, C39D mutant exhibited about twofold decline in inhibitory efficiency compared to RmRec (Figure 7B). Under these conditions, dRec inhibited GRK1 by almost 70%, which is close to the effect of RmRec. Meanwhile, the differences between the behavior of dRec and RmRec were absent only at their saturating levels (40 μM recoverin monomer), whereas at fourfold lower concentration disulfide dimer inhibited GRK1 one third less efficiently than reduced monomer (data not shown). In the absence of Ca^2+^, neither RmRec nor C39D exhibited significant influence on GRK1 activity. Meanwhile, dRec inhibited the enzyme by 40%, thereby demonstrating constitutive Ca^2+^-independent activity toward the enzyme (Figure 7B). Notably, the incubation of dRec with excess of DTT almost completely restored normal regulatory activity of recoverin toward GRK1 (data not shown) indicating that disulfide dimerization of recoverin is functionally reversible.

To examine whether the altered GRK1 regulation observed in the case of dRec and C39D mutant resulted from changes in recoverin affinity to GRK1, we employed SPR spectroscopy to monitor recoverin interaction with N-terminal domain of the enzyme (N-GRK1) or its fragment M1-S25 (1-25GRK1), which both contain specific recoverin-binding site (Ames et al., 2006). To this end, N-GRK1 or 1-25GRK1 were immobilized on the surface of SPR sensor chip by amine coupling and a set of injections of RmRec, dRec, OmRec and C39D mutant was carried out. The SPR sensograms were approximated by the heterogeneous ligand model (1) yielding respective equilibrium constants (Table 3). In the presence of calcium, the affinity of N-GRK1 to the three recoverin variants was 6- to 10-fold lower than to RmRec. This effect was less noticeable for their binding to 1-25GRK1. The binding constants for dRec interaction with 1-25RK and N-GRK1 are close to each other, whereas the affinity of OmRec and C39D to 1-25GRK1 is about 1.7- to 2-fold higher than to N-GRK1. Meanwhile, in the absence of calcium, none of the recoverin forms shows noticeable binding to the GRK1 fragments (data not shown). Thus, the revealed ability of Ca^2+^-free dRec to inhibit GRK1 may be due to its interaction with a site located outside the N-terminal domain of the enzyme or can be related to increased membrane affinity of the apo-dimer.

**Table 3:** The affinity of Ca^2+^-bound recoverin variants to N-terminal domain of GRK1 (N-GRK1) and its fragment corresponding to the residues M1-S25 (1-25GRK1), estimated using SPR spectroscopy according to the heterogeneous ligand model (1).

Ligand	*K*_D_, M			
	RmRec	OmRec	C39D	dRec
N-GRK1	(5.35 ± 0.38) × 10^-6^	(3.13 ± 0.11) × 10^-5^	(5.02 ± 0.11) × 10^-5^	(3.13 ± 0.48) × 10^-5^
1-25GRK1	(1.05 ± 0.06) × 10^-5^	(1.60 ± 0.67) × 10^-5^	(3.13 ± 0.14) × 10^-5^	(3.12 ± 3.08) × 10^-5^

## Discussion

Recoverin is one of a few photoreceptor proteins demonstrating thiol oxidation in response to moderate doses of light illumination. Since intravitreous administration of anti-recoverin antibodies induces in rats photoreceptor dysfunction similar to that produced by excessive light illumination (Maeda et al., 2001; Ohguro et al., 2001), recoverin is likely involved in PD mechanisms. Therefore, we aimed at identification of the oxidized forms of recoverin accumulating in mammalian retina exposed to different doses of light illumination and exploration of structural and functional properties of these forms. The first model of retinal PD employed pigmented rabbits, since they share with humans many features of ocular anatomy, including eyeball size, its internal structure, and optical system along with biochemical features (Zernii et al., 2016). We developed rabbit model of iatrogenic light-induced retinal damage: the restrained animals were exposed to short-term (3 h) illuminations by visual light of either moderate (halogen lamp, 2,200 lx, 0.011 W/cm^2^) or high intensity (halogen lamp, 30,000 lx, 0.15 W/cm^2^). The latter irradiation dose corresponded to that received by the retina from common operative microscopes during 0.5–1 h in the course of, for instance, keratoplasty or vitrectomy [(Irvine et al., 1984), our own calculations]. In the second model of retinal PD, the long-term low-intensity illumination was applied to unrestrained rats: the rats were exposed to 14 h illuminations by visual light of moderate intensity (metal halide lamp, 2,500 lx, 0.003 W/cm^2^), similarly to conditions described in a number of previous studies (van Norren and Gorgels, 2011; Novikova et al., 2014).

Analysis of recoverin fractions extracted from the light-exposed eyes of the both species revealed disulfide dimerization of recoverin. Fraction of the dimer found in the dark-adapted retinas was substantially lower. In the rabbit model, illumination dose-dependent accumulation of the dimeric recoverin was observed, in accord with our previous findings (Zernii et al., 2015b). The disulfide dimerization of recoverin in rat retinas was discovered for the first time. Unexpectedly, the retina of control rats contained relatively high basic level of recoverin dimer (∼40%) and we revealed only moderate increase of the dimer content upon illumination. The enhanced oxidation of recoverin in the control group can be related to higher basic redox potential in the retina of albino rats as compared to pigmented rabbits (Sohal and Weindruch, 1996). It is possible that the content of recoverin dimer transiently increased even above this basic level, but decayed by the completion of the long-term illumination, characteristic to the rat model. Previous proteomic study of rats identified arrestin as the only retinal protein exhibiting the light-dependent disulfide dimerization (Lieven et al., 2012). The failure to detect dRec in that work may be due to about two orders of magnitude lower amount of recoverin in retina compared to arrestin (Klenchin et al., 1995; Vishnivetskiy et al., 2007) along with lower sensitivity of 2D electrophoresis relative to Western blotting with ECL detection used here. In contrast to rats, the light-induced disulfide dimerization of recoverin in the rabbit retina was accompanied by accumulation of its monomer with an intramolecular disulfide bond and disulfide multimers/aggregates, depending on intensity of illumination. Indeed, in contrast to rat recoverin with single Cys residue, the rabbit protein contains two Cys residues (C29 and C39) enabling formation of an intramolecular disulfide bridge. The second Cys residue seems to be involved into accumulation of the disulfide-linked recoverin multimers/aggregates. Thus, formation of intramolecular disulfides and disulfide multimerization of recoverin might be inherent to its orthologs containing the second Cys residue (rabbit, human, etc.). Notably, MALDI mass-spectrometry analysis of rabbit recoverin samples obtained under the high-intensity light illumination (0.15 W/cm^2^) revealed another monomer of the protein with two extra oxygen atoms. Despite lack of strict MS evidences, we attributed this recoverin form to its derivative with C39 converted into sulfinic acid. Firstly, peptide mass fingerprinting of the *in vivo* oxidized rabbit recoverin demonstrated that neither of other redox-sensitive residues (M, W, H) were oxidized. Thus, C39 is the most redox sensitive residue of recoverin, due to its low pK_a_ value favoring formation at physiological pH of the highly reactive thiolate anion (Permyakov et al., 2007). Secondly, MS and MS/MS analysis of *in vitro* oxidized recombinant bovine recoverin demonstrated stepwise oxidation of C39 with formation of sulfenic and sulfinic acids. Finally, the conversion of C39 of recoverin into sulfenic acid under mild oxidizing conditions was previously confirmed by X-ray crystallography (Ranaghan et al., 2013). Overall, we propose that the oxidation state of recoverin depends on regime of the visual light illumination: low-intensity exposure yields accumulation of recoverin forms with an intermolecular or intramolecular disulfide bond, whereas high-intensity illumination apparently results in further oxidation of the thiol with accumulation of sulfinic acid.

The histological studies of both rat and rabbit models indicate that thiol oxidation of recoverin preceded or accompanied photoreceptor cells death via apoptosis, but kinetic parameters of the apoptotic changes were species-dependent. In the rat model, despite the lower light intensity (0.011 W/cm^2^) the signs of acute apoptosis such as karyopyknosis and formation of apoptotic bodies (Noell et al., 1966; Novikova et al., 2014) manifested in some regions of the retina immediately after the exposure. Furthermore, they were accompanied by shift of the photoreceptor cell bodies in the vitreal direction that is commonly observed in the final step of photoreceptor apoptosis (Marc et al., 2008). By contrast, rabbit retina after illumination by the high-intensity light (0.15 W/cm^2^) exhibited no signs of photoreceptor death. The photo-induced lesions could develop in the retina regions beyond the scope of our analysis, since at early stages the damage is of focal nature (Mckechnie and Foulds, 1981). Nevertheless, the multifocal histological picture of acute photoreceptor apoptosis [loss of outer and inner segments, karyopyknosis (Hoppeler et al., 1988)], accompanied by general changes in the retina cytoarchitecture, was observed only on the third day post-exposure. The revealed discrepancies between rat and rabbit models could be related to higher susceptibility of albino rats to retinal PD (van Norren and Gorgels, 2011; Hunter et al., 2012). In addition, they could be related to differences in the time intervals between the illumination onset and histological analysis, used in the rat and rabbit models (14 h versus 3 h, respectively). It seems that 3 h had been insufficient for development of photoreceptor damage in rabbits, since we observed no early signs of photoreceptor deterioration, such as swelling and shortening of their outer segments, but these effects were previously observed is response to low-intensity light for 5 h (Zernii et al., 2015b). Thus, rabbit retina required at least 5 h to develop well-defined response to visual light irradiation, regardless of its intensity.

The low-intensity light illumination (0.011 W/cm^2^) did not cause any morphological alterations in the rabbit retina neither immediately after the exposure nor after 14 subsequent days. This is in agreement with the previous report that 4-h exposure of the rabbit retina to light illumination of at least 0.045 W/cm^2^ is required for induction of noticeable retinal damage (Lawwill, 1973). Thus, visible light causes PD of photoreceptors only upon reaching of a certain threshold illumination dose (van Norren and Gorgels, 2011), and that the light-induced destructive processes in photoreceptors may be delayed, often developing in the cells that exhibit normal morphology by the time of completion of the light exposure. This suggests existence in photoreceptor cells of certain mechanisms for making life-death decisions, which depend on severity of the developing light-induced oxidative stress and likely involve specific redox-sensitive proteins, such as recoverin.

The low-intensity illumination of rabbit retina induced only moderate increase in the content of the disulfide dimer, which likely did not promote photoreceptor apoptosis. Meanwhile, the high-intensity illumination of rabbit eyes induced accumulation of the recoverin dimer and its aggregates apparently along with C39 oxidation up to sulfinic acid, accompanied by photoreceptor death via apoptosis. In the rat model, the dimer to monomer ratio also increased upon illumination, but to a lesser extent, despite the fact that photoreceptors had more time for response to the light-induced stress (14 h versus 3 h in the rabbit model). We suppose that excess of the thiol-oxidized proteins, such as recoverin, was utilized in proteasome (Shang and Taylor, 2012), as confirmed by formation of mixed disulfides of recoverin with proteasome subunits upon the prolonged low-intensity illumination (Zernii et al., 2015b). Thus, photoreceptor apoptosis seems to have been induced under pronounced accumulation of disulfide dimer of recoverin and C39 oxidation to sulfinic acid. Given the existence of several mechanisms for photoreceptor death induction (Hao et al., 2002; Chen et al., 2016), its plausible that different combinations of recoverin thiol oxidation could trigger different mechanisms of cell death.

Our *in vitro* studies indicate that disulfide dimerization of recoverin and formation of its oxidized monomer differently affect secondary and tertiary structures of the protein, especially in the absence of calcium. α-Helicity and thermal stability of the Ca^2+^-free recoverin forms decreased in the following order: reduced monomer – oxidized monomer – disulfide dimer. Notably, *T*_1/2_ for thermal unfolding of apo-OmRec was similar to that for the C39D mutant that mimics conversion of C39 into sulfenic/sulfinic acid (Permyakov et al., 2012), indicating importance of electrostatic charge in 39th position of the protein for maintenance of its structural stability. Meanwhile, calcium binding stabilized secondary and tertiary structures of OmRec and C39D mutant, making them closer to Ca^2+^-bound RmRec. Consistently, in the presence of calcium tertiary structures of the sulfenate-containing monomer of recoverin and its C39D mutant were equivalent to that of the reduced protein (Ranaghan et al., 2013). Despite partial structural destabilization of the Ca^2+^-free forms of C39D and dRec, both of them exhibited common thermal denaturation profile (Figure 6) and fluorescence spectrum (data not shown) and remained responsive to Ca^2+^ (Figures 5, 6) indicating preservation of general fold of recoverin. Furthermore, they even demonstrated slightly elevated Ca^2+^ affinity (Table 1). The thiol oxidation-induced destabilization of Ca^2+^-free recoverin with myristoyl group sequestered in the hydrophobic pocket seemed to facilitate the conversion into the Ca^2+^-bound state, in which myristoyl group and non-polar residues of the pocket are solvent-exposed (for review, see Ames and Lim, 2012). This effect was most pronounced in the case of dRec suggesting that myristate and hydrophobic pocket may have been partially solvent exposed in the Ca^2+^-free dimer. Consistently, our studies employing hydrophobic probe bis-ANS showed markedly increased surface hydrophobicity of dRec, apo-form of which was reminiscent of Ca^2+^-saturated reduced recoverin in this respect.

Since myristoyl group and the hydrophobic pocket residues of recoverin are responsible for its binding to photoreceptor membranes and GRK1, respectively (for review, see Ames and Lim, 2012), disulfide dimer of recoverin was expected to exhibit these functional activities in the absence of calcium. Indeed, Ca^2+^-free dRec demonstrated approximately twofold increase in the membrane-bound fraction and inhibition of the enzyme by 40%, showing its partial constitutive activity. The activity of Ca^2+^-free dRec toward GRK1 could be due to their interaction via the GRK1 site located outside of its N-terminal domain, or could be related to increased membrane affinity of the dimer. Similar effect was recently observed in the case of recoverin chimera with C-terminal segment of GCAP2, which in Ca^2+^-free conformation exhibited elevated membrane and GRK1 binding (Zernii et al., 2015a).

**FIGURE 8:**
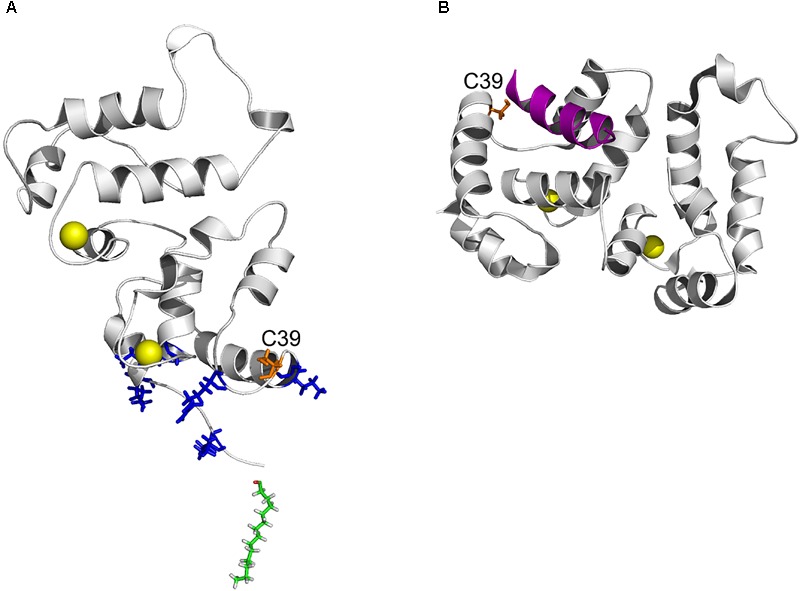
Position of C39 in three-dimensional structure of recoverin bound to membrane and GRK1. **(A)** Topology of recoverin on membrane surface built based on NMR structures of myristoylated Ca^2+^-bound protein [PDB entry 1JSA (Ames et al., 1997; Valentine et al., 2003)]. C39 (orange), calcium ions (yellow), myristoyl residue (green) and the basic residues (K5, K11, K37, R43, and K84) in close contact with the membrane are indicated. **(B)** The structure of recoverin complex with GRK1 [PDB entry 2I94 (Ames et al., 2006)]. N-terminal amphipathic helix of GRK1 (magenta), calcium ions (yellow) and C39 (orange) are indicated. The images were created using PyMol Molecular Graphics System v.1.4.1 (Schrödinger, LLC).

The increased hydrophobicity of Ca^2+^-bound dRec (Figure 6D) might have accounted for its high susceptibility to aggregation (Zernii et al., 2015b) that would reduce fraction of the functional protein capable of binding to membranes (Figure 7A). In addition, this reduction could be associated with alterations in recoverin-membrane interface in the case of dRec due to modification of C39 by the second recoverin molecule. The structure of Ca^2+^-saturated dRec also seemed to be not optimal for specific binding of GRK1 (Table 3). In contrast to dRec, OmRec and C39D mutant underwent more limited structural changes and their apo-forms remained non-functional. However, in the presence of calcium the membrane binding and regulatory activity of these forms were strongly suppressed (Figure 7B) (Permyakov et al., 2012; Ranaghan et al., 2013). We attribute this phenomenon to direct effect of the negative charge introduced into 39th position of recoverin. In the Ca^2+^-loaded protein bound to membrane C39 residue is positioned in close proximity to the negatively charged surface of phospholipid bilayer (Figure 8A), whereas in the recoverin-GRK1 complex this residue flanks amphipathic N-terminal α-helix of the enzyme (Figure 8B). Thus, the introduction of negative charge into position 39 of recoverin may have perturbed these interactions. Consistently, our SPR measurements and previous pull-down studies revealed that affinity of Ca^2+^-bound OmRec and C39D mutant to GRK1 was substantially decreased as compared to reduced recoverin (Table 3) (Ranaghan et al., 2013).

Overall, the functional activities of recoverin were clearly modulated by redox state of its conservative cysteine residue in a Ca^2+^-dependent manner. The thiol-oxidized monomer of recoverin demonstrated impaired ability to bind photoreceptor membranes and inhibit GRK1 regardless of calcium level. In contrast, disulfide dimer of the protein exhibited lowered functional activity in the Ca^2+^-saturated form, but acquired partial activity in the absence of calcium. It should be added that the incubation of dRec with excess of DTT restored normal membrane binding and regulatory activity of recoverin indicating that its disulfide dimerization is functionally reversible.

**FIGURE 9:**
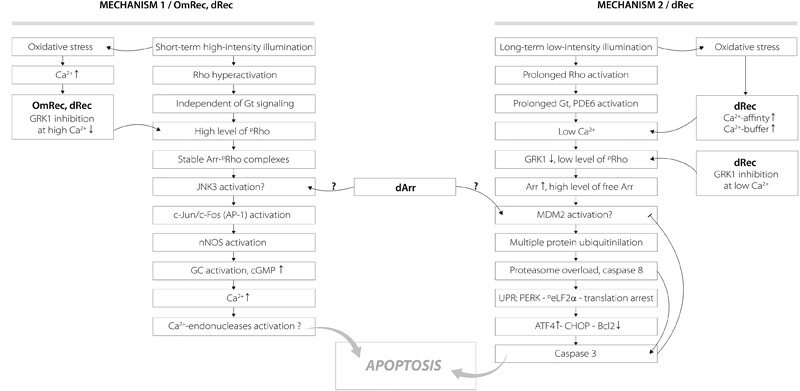
Hypothetical scheme describing potential roles of disulfide dimer and thiol-oxidized monomer of recoverin in the mechanisms of photoreceptor apoptosis induced by visual light. The designations are as follows: Arr, arrestin; dArr, disulfide dimer of arrestin; GRK1, G-protein coupled kinase-1; Gt, transducin; PDE6, rod cGMP-specific 3’,5’-cyclic phosphodiesterase; Rho, rhodopsin; ^p^Rho, phosphorylated rhodopsin; OmRec, monomeric recoverin with C39 converted into sulfinic acid; dRec, disulfide dimer of recoverin; JNK3, c-Jun N-terminal kinase 3; c-Jun/c-Fos (AP-1), activator protein 1 – heterodimeric transcription factor; nNOS, neuronal nitric oxide synthase 1; GC, guanylate cyclase; MDM2, mouse double minute 2 homolog – E3 ubiquitin-protein ligase; UPR, unfolded protein response; CHOP, C/EBP homologous protein – pro-apoptotic transcription factor; PERK, protein kinase R (PKR)-like endoplasmic reticulum kinase – translation initiation factor 2-alpha kinase 3; ^p^eLF2α, phosphorylated translation initiation factor 2α; ATF4, activating transcription factor 4; Bcl2, B-cell lymphoma 2 – apoptosis regulator protein. For details, refer to section “Discussion.”

The revealed oxidation-induced alterations in functional properties of recoverin may affect physiological activity of the protein contributing to the light-induced retinal damage, triggered via two mechanisms depending on intensity of the light illumination (Figure 9) (Hao et al., 2002; Wang and Chen, 2014). We propose that disulfide dimer and thiol-oxidized monomer of recoverin play specific roles in progression of these mechanisms. The retinal damage caused by the short-term high-intensity illumination (mechanism 1, Figure 9) is associated with significantly reduced kinetics of rhodopsin dephosphorylation (Ishikawa et al., 2006). The excessive phosphorylation of the receptor would induce formation of stable arrestin-rhodopsin complexes (Vishnivetskiy et al., 2007), accumulation of which is known to enhance retinal degeneration (Chen et al., 2006). Under the oxidative stress induced by intense illumination some fraction of recoverin is expected to be converted into the thiol-oxidized monomer (Figure 2) that exhibits diminished binding to photoreceptor membranes and GRK1 inhibition at high calcium level (Figure 7). Besides, marked fraction of recoverin would form disulfide dimer with reduced affinity to membranes and GRK1 in the presence of calcium (Figure 7A and Table 3). Notably, one of the early events accompanying high-intensity light-induced oxidative stress and preceding photoreceptor apoptosis is a pronounced increase in intracellular calcium level through activation of nNOS and NO-sensitive guanylate cyclase (Donovan et al., 2001). Thus, the C39 oxidation of recoverin should suppress its activity, thereby promoting excessive phosphorylation of the receptor, favoring formation of arrestin-rhodopsin complexes [arrestin is accumulated in light-exposed ROS (Broekhuyse et al., 1985)] and development of the respective retinal damage. Besides, the malfunction of the oxidized Ca^2+^-bound recoverin could contribute to photoreceptor degeneration in the post-exposure period, when the retina is reared under dark or cyclic light conditions (Organisciak and Vaughan, 2010). These effects are similar to the photoreceptor degeneration in the rat model of cancer-associated retinopathy produced by intravitreous administration of anti-recoverin antibodies, which suppresses recoverin functioning and thereby promotes rhodopsin phosphorylation (Ohguro et al., 1999, 2001; Maeda et al., 2001). Furthermore, the mouse phenotypes overexpressing GRK1 (GRK1 upregulation is analogous to recoverin inactivation) exhibit an increased susceptibility to high-intensity illumination (Whitcomb et al., 2010). The mechanism underlying the induction of photoreceptor apoptosis by arrestin-rhodopsin complexes remains poorly understood. We speculate that it may involve one of the protein targets of visual arrestin, JNK3 (Song et al., 2006), which is able to upregulate pro-apoptotic factor AP-1 (Coffey et al., 2002). Yet, AP-1 induction by high-intensity light was previously observed not only in wild-type animals, but also in the phenotypes lacking GRK1 or arrestin (Hao et al., 2002). AP-1 can activate downstream pro-apoptotic proteins, such as nNOS that can be upregulated by c-jun, a component of AP-1 complex (Cheng et al., 2014). Upregulation of nNOS and NO-sensitive guanylate cyclase would further increase calcium influx into the photoreceptors through cGMP-gated channels and promote their apoptosis through mitochondrial depolarization and, likely, activation of Ca^2+^-dependent endonucleases (Donovan et al., 2001).

Opposite to the thiol-oxidized monomer of recoverin signs of which were found only in the retinas exposed to high-intensity illumination, noticeable accumulation of disulfide dimer of the protein occurs during both high-intensity and low-intensity light illumination (Figure 1). At low calcium level, corresponding to the light-adapted photoreceptor cells, significant fraction of dRec could remain on the membranes and inhibit GRK1 (Figure 7). This would prevent rhodopsin from desensitization, thereby supporting the light-induced oxidative stress. These considerations are especially relevant to the retinal damage induced by long-term low-intensity light illumination (mechanism 2, Figure 9). The GRK1 downregulation observed under these conditions (Hajkova et al., 2010) should synergistically support the inhibitory action of Ca^2+^-free dRec. The resulting activation of Gt by rhodopsin and operation of the phototransduction cascade involve constantly active PDE6 and lowered intracellular calcium level (Wang and Chen, 2014). The latter could be supported by dRec due to the Ca^2+^ buffering function of recoverin (Makino et al., 2004) and increased Ca^2+^ affinity of dRec (Figure 5). The subsequent excessive cGMP utilization is suggested to induce metabolic stress, followed by multiple protein ubiquitination, proteasome overload and induction of apoptosis via PERK pathway of the unfolded protein response and activation of caspase 3 (Wang and Chen, 2014). Consistently, recoverin forms disulfide complexes with proteasome subunits upon prolonged exposure to low-intensity light (Zernii et al., 2015b). The multiple ubiquitination of photoreceptor proteins could be ensured by photoreceptor MDM2, an ubiquitin ligase that was recognized as a protein target of visual arrestin (Song et al., 2006). Interestingly, arrestin preferentially binds MDM2 being in the basal (receptor-free) conformation (Song et al., 2006). Free arrestin might be accumulated in the outer segments only after translocation from the inner segments in response to the low-intensity light illumination (Hajkova et al., 2010), and when rhodopsin phosphorylation is suppressed as in the case of GRK1 inhibition by Ca^2+^-free dRec. Such hypothetic regulation of MDM2 by arrestin may be affected by the light-induced disulfide dimerization of the latter (Lieven et al., 2012; Zernii et al., 2015b). Notably, the disulfide dimerization would also reduce affinity of recoverin to membranes and GRK1 at high calcium levels (Figure 7A and Table 3), corresponding to the dark-adapted photoreceptor cells. This effect could contribute to photoreceptor degeneration in post-exposure period, when the retina is reared under dark or cyclic light conditions (Organisciak and Vaughan, 2010).

The conservative thiols are functionally important for some other proteins of the NCS family. For instance, substitutions of the Cys residue in photoreceptor proteins GCAP1 (C29) and GCAP2 (C35) suppress their activity (Ermilov et al., 2001; Hwang et al., 2004). Oxidative stress triggers disulfide dimerization of a non-photoreceptor NCS protein, visinin-like protein 1 (VILIP-1) (Chen et al., 2009; Liebl et al., 2014). Interestingly, VILIP-1 oxidation involves not its conservative Cys (C38) corresponding to C39 of recoverin, but the C-terminal residue C187, which is responsible for Ca^2+^-dependent disulfide dimerization of the protein (Chen et al., 2009; Liebl et al., 2014). Functional significance of this process is unclear, but it affects regulatory activity of the protein toward guanylate cyclase B (Chen et al., 2009). Furthermore, VILIP-1 oxidation is associated with amyotrophic lateral sclerosis (ALS), since VILIP-1 is a part of the ALS-specific protein aggregates, whereas its soluble dimers are enriched in spinal cord of the animals with experimental ALS and their formation generally correlates with the disease progression (Liebl et al., 2014).

Overall, considering conservative nature of the cysteine residues in NCS proteins, one can expect that redox sensitivity is inherent to members of this family. The thiol-oxidized forms of these proteins and functional significance of such modifications remain to be established. Since alterations in redox state of NCS proteins may contribute to progression of the oxidative stress-related neurological and neuro-ophthalmological diseases, the use of broad spectrum antioxidants or specific sulfur reductants might be regarded as a promising approach to their treatment (Organisciak and Vaughan, 2010; Lieven et al., 2012).

## Author Contributions

EZ, PP, and SP participated in the design of the study and performed literature screening and analysis. OG, NT, and EZ developed animal models of light-induced retinal degeneration. NT and EZ performed immunoaffinity chromatography and immunoblotting. MS conducted mass-spectrometry studies. OG performed histological examinations. AN, EN, VV, and DZ prepared recoverin forms. IS performed calcium binding assay. AN and EN conducted fluorescence and CD measurements. VB and EZ performed membrane binding (equilibrium centrifugation) and rhodopsin phosphorylation assays. AK performed SPR analysis. EN, EZ, and SP wrote the manuscript.

## Conflict of Interest Statement

The authors declare that the research was conducted in the absence of any commercial or financial relationships that could be construed as a potential conflict of interest.

## Supplementary Material

FIGURE S1 MS/MS spectra of recoverin peptides E38-R43 **(A)** and F23-R43 **(B)** containing C39 in reduced form (upper panel) or oxidized with formation of sulfinic acid (lower panel). The identified amino acid sequences of the peptides are indicated in red.

Table S1 The results of peptide mass fingerprinting of recoverin samples extracted from the light-illuminated (metal halide lamp, 2,500 lx, 14 h) *in vivo* rat retinas.

Table S2 The results of peptide mass fingerprinting (MS and MS/MS) of recoverin samples extracted from the rabbit retinas illuminated *in vivo* according to the scheme 1 (halogen lamp, 2,200 lx, 3 h).

## Abbreviations

AMD: age-related macular degeneration
bis-ANS: 4,4’-dianilino-1,1’-binaphthyl-5,5’-disulfonic acid
CD: circular dichroism
C39D: recoverin mutant with C39 replaced by aspartic acid
DTT: dithiothreitol
dRec: disulfide dimer of recombinant wild type recoverin
EDTA: ethylenediaminetetraacetic acid
EGTA: ethylene glycol-bis(2-aminoethylether)-N,N,N’,N’-tetraacetic acid
1-25GRK1: N-terminal fragment of GRK1 (amino acids M1-S25) fused with glutathione-*S*-transferase
HEPES: *N*-(2-hydroxyethyl)piperazine-N0-(2-ethanesulfonic acid)
λ: position of fluorescence spectrum maximum
NCS: neuronal calcium sensor
N-GRK1: N-terminal domain of GRK1 (amino acids Met1-Gly183) fused with glutathione-*S*-transferase
nNOS: neuronal nitric oxide synthase
OmRec: oxidized monomer of recombinant wild type recoverin
PD: photochemical damage
RmRec: reduced monomer of recombinant wild type recoverin
ROS: rod outer segments
RPE: retinal pigment epithelium
SPR: surface plasmon resonance
*T*_1/2_: mid-transition temperature
Tris: tris(hydroxymethyl) aminomethane
