# Supplementary figures and images for: Light-Induced Thiol Oxidation of Recoverin Affects Rhodopsin Desensitization
Source: Front Mol Neurosci. 2019 Jan 7;11:474. doi: 10.3389/fnmol.2018.00474 (PMC6330308; doi:10.3389/fnmol.2018.00474)

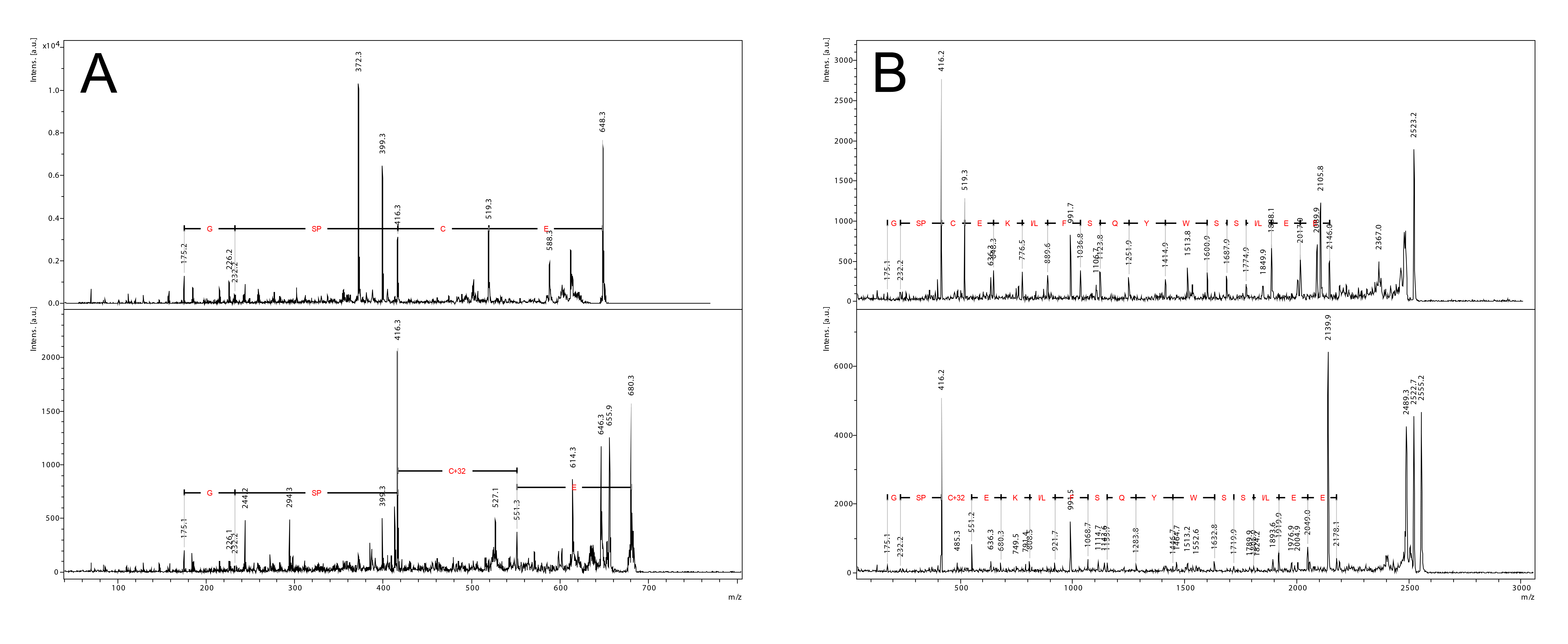

Supplement: FIGURE S1 — MS/MS spectra of recoverin peptides E38-R43 (A) and F23-R43 (B) containing C39 in reduced form (upper panel) or oxidized with formation of sulfinic acid (lower panel). The identified amino acid sequences of the peptides are indicated in red. [file Image_1.TIF]
