# Supplementary material for: Light-Induced Thiol Oxidation of Recoverin Affects Rhodopsin Desensitization
Source: Front Mol Neurosci. 2019 Jan 7;11:474. doi: 10.3389/fnmol.2018.00474 (PMC6330308; doi:10.3389/fnmol.2018.00474)
Supplement: Table S1 — The results of peptide mass fingerprinting of recoverin samples extracted from the light-illuminated (metal halide lamp, 2,500 lx, 14 h) in vivo rat retinas. [file Table_1.DOC]

| **Table S1. The results of peptide mass fingerprinting of recoverin samples extracted from the light-illuminated (metal halide lamp, 2,500 lx, 14 h) *in vivo* rat retinas.** | | |
| --- | --- | --- |
| **Position** | **m/z** | **Peptide** |
| 155 - 161 | 868.4732 | K.IWAFFGK.K |
| 64 - 71 | 991.5144 | K.AYAQHVFR.S |
| 155 - 162 | 996.5717 | K.IWAFFGKK.D |
| 185 - 192 | 1002.5630 | R.LIQFEPQK.V |
| 185 - 194 | 1229.7309 | R.LIQFEPQKVK.E |
| 152 - 162 | 1324.7372 | R.AEKIWAFFGKK.D |
| 140 - 151 | 1427.6855 | K.NLPDDENTPEKR.A |
| 44 - 55 | 1500.7749 | R.ITRQEFESIYSK.F |
| 185 - 196 | 1514.8680 | R.LIQFEPQKVKER.I |
| 56 - 71 | 1924.9529 | K.FFPDSDPKAYAQHVFR.S |
| 163 - 180 | 2066.9793 | K.DDDKLTEEEFIEGTLANK.E |
| 23 - 43 | 2507.1666 | K.FTEEELSAWYQSFLKECPSGR.I* |
| 163 - 184 | 2578.2954 | K.DDDKLTEEEFIEGTLANKEILR.L |
| 72 - 101 | 3285.6164 | R.SFDANSDGTLDFKEYVIALHMTTAGKPTQK.L |
| 12 - 43 | 3817.8752 | K.EILEELQLNTKFTEEELSAWYQSFLKECPSGR.I* |
| *Cys-containing peptides | | |
|  | | |
