# Supplementary material for: Light-Induced Thiol Oxidation of Recoverin Affects Rhodopsin Desensitization
Source: Front Mol Neurosci. 2019 Jan 7;11:474. doi: 10.3389/fnmol.2018.00474 (PMC6330308; doi:10.3389/fnmol.2018.00474)
Supplement: Table S2 — The results of peptide mass fingerprinting (MS and MS/MS) of recoverin samples extracted from the rabbit retinas illuminated in vivo according to the scheme 1 (halogen lamp, 2,200 lx, 3 h). [file Table_2.DOC]

| **Table S2. The results of peptide mass fingerprinting (MS and MS/MS) of recoverin samples extracted from the rabbit retinas illuminated *in vivo* according to the scheme 1 (halogen lamp, 2,200 lx, 3h).** | | |
| --- | --- | --- |
| **Position** | **m/z** | **Peptide** |
| 2–5 | 615.4297 | M.GNSK.S + Myristoyl (N-term G)* |
| 38–43 | 648.2951 | K.ECPSGR.I* |
| 155–161 | 854.4489 | K.IWGFFGK.K |
| 56–63 | 936.4395 | K.FFPDADPK.A |
| 64–71 | 991.5116 | K.AYAQHVFR.S |
| 185–192 | 1002.5528 | R.LIQFEPQK.V* |
| 47–55 | 1114.5263 | R.QEFESIYAK.F |
| 12–22 | 1315.7009 | K.EILEDLQLNTK.F |
| 120–131 | 1405.7658 | K.NEVLEIVMAIFK.M |
| 72–84 | 1416.6293 | R.SFDANSDGTLDFK.E |
| 140–151 | 1450.6979 | K.HLPDDENTPEKR.T |
| 44–55 | 1484.7914 | R.ITRQEFESIYAK.F |
| 85–101 | 1874.0024 | K.EYVIALHMTTAGKPSQK.L |
| 23–37 | 1923.8839 | K.FTEEELCTWYQSFLK.E |
| 102–119 | 1984.9571 | K.LEWAFSLYDVDGNGAISK.N |
| 163–180 | 2084.9190 | K.DDDKLTEEEFIEGTMANK.E |
| 162–180 | 2213.0255 | K.KDDDKLTEEEFIEGTMANK.E |
| 23–43 | 2551.1380 | K.FTEEELCTWYQSFLKECPSGR.I + 2 Dehydro(C)* |
| 72–101 | 3271.5890 | R.SFDANSDGTLDFKEYVIALHMTTAGKPSQK.L* |
| 44–71 | 3374.5628 | R.ITRQEFESIYAKFFPDADPKAYAQHVFR.S |
| *Peptide sequence was confirmed by MS/MS analysis. | | |
